# Preoperative analysis of factors associated with prolonged pneumoretroperitoneum time during retroperitoneal laparoscopic nephroureterectomy for upper tract urothelial carcinoma

**DOI:** 10.1186/s12894-024-01538-0

**Published:** 2024-07-29

**Authors:** Masato Yanagi, Tomonari Kiriyama, Jun Akatsuka, Yuki Endo, Hayato Takeda, Tsutomu Hamasaki, Taiji Nishimura, Yukihiro Kondo

**Affiliations:** 1 Department of Urology, Nippon Medical School Hospital, 1-1-5, Sendagi, Bunkyo-Ku, Tokyo, 113-8603 Japan; 2 Department of Radiology, Nippon Medical School Hospital, 1-1-5, Sendagi, Bunkyo-Ku, Tokyo, 113-8603 Japan; 3 Department of Urology, Nippon Medical School Musashikosugi Hospital, 1-396, Kosugimachi, Nakahara-Ku, Kawasaki-City, Kanagawa 211-8533 Japan

**Keywords:** Body mass index, Perirenal fat stranding, Perirenal fat thickness, Pneumoretroperitoneum time, Retroperitoneoscopic nephroureterectomy, Upper tract urothelial carcinoma

## Abstract

**Background:**

Prolonged laparoscopic nephroureterectomy (LNU) for upper tract urothelial cancer (UTUC) can increase the frequency of intravesical recurrence after surgery. Therefore, it is important for urological surgeons to have knowledge on preoperative risk factors for prolonged LNU. However, few studies have investigated the risk factors for prolonged LNU. We hypothesized that the quantity of perirenal fat affects the pneumoretroperitoneum time (PRT) of retroperitoneal LNU (rLNU). This study aimed to investigate the preoperative risk factors for prolonged PRT during rLNU.

**Methods:**

We reviewed the data of 115 patients who underwent rLNU for UTUC between 2013 and 2021. The perirenal fat thickness (PFT) observed on preoperative computed tomography (CT) images was used to evaluate the perinephric fat quantity. Preoperative risk factors for PRT during rLNU were analyzed using logistic regression models. The cutoff value for PRT was determined based on the median time.The cutoff values for fat-related factors influencing PRT were defined according to receiver operating characteristic curve analysis.

**Results:**

The median PRT for rLNU was 182 min (interquartile range, 155–230 min). The cutoff values of posterior, lateral, and anterior PFTs were 15 mm, 24 mm, and 6 mm, respectively. Multivariate analysis revealed that a posterior PFT ≥ 15 mm (odds ratio [OR], 2.72; 95% confidence interval, 1.04–7.08; *p* = 0.0410) was an independent risk factor for prolonged PRT.

**Conclusions:**

Thick posterior PFT is a preoperative risk factor for prolonged PRT during rLNU. For patients with UTUC and thick posterior PFT, surgeons should develop optimal surgical strategies, including the selecting an expert surgeon as a primary surgeon and the selecting transperitoneal approach to surgery or open surgery.

## Background

Upper tract urothelial cancer (UTUC) is a relatively uncommon condition that accounts for 5 to 10% of all urothelial malignancies [1]. Although nephroureterectomy (NU) with bladder cuff excision is the gold standard treatment for non-metastatic UTUC, laparoscopic nephroureterectomy (LNU) is performed in patients with UTUC worldwide [2]. However, one of the major concerns associated with NU is intravesical recurrence (IVR). Interestingly, some studies have reported that prolonged pneumoperitoneum time of transperitoneal LNU and pnemoretroperitoneum time (PRT) of retroperitoneal LNU (rLNU) are risk factors for IVR after surgery; furthermore, these studies have speculated that long CO_2_ gas pressure times resulted in intraluminal seeding of cancer cells to the bladder [3, 4]. Additionally, prolonged surgical times could result in surgical team fatigue, increase the likelihood of technical errors, and increase perioperative complications, such as pulmonary embolism and rhabdomyolysis [5, 6]. Accordingly, it is important for urological surgeons to be aware of the preoperative risk factors for prolonged laparoscopy times during LNU when determining surgical strategies because these times can affect oncological outcomes and perioperative complications. However, few studies have investigated preoperative risk factors for prolonged laparoscopy times during LNU.

Perirenal fat is the one that encapsulates the kidney and fills out the space between the kidney and the adjacent retroperitoneal tissue, renal parenchyma, and adrenal glands [7]. It is surrounded by a complete renal fascia with complete system of blood supply, lymphatic fluid drainage, and innervation [8]. It is adjacent to the kidneys, active in metabolism and adipokine secretion, and shares the same developmental origin as the typical visceral fat [9]. Visceral obesity has a detrimental impact on surgery. Perirenal fat thickness (PFT), an indirect indicator of visceral obesity, has been identified as an independent predictor of postoperative complications in surgeries for gastric and colorectal cancers [10, 11]. Severe complications such as anastomotic leakage are believed to result from challenging dissections caused by excessive visceral fat and a restricted operative field [10, 11]. Additionally, thick perirenal fat has been associated with prolonged operating times and increased intraoperative blood loss during transperitoneal laparoscopic adrenalectomy [12]. In addition, it was reported that the amount of perirenal fat, especially that of anterior perirenal fat, was correlated with the operative time during laparoscopic donor nephrectomy using transperitoneal approach [13]. Moreover, there was a case report of rLNU that had to be converted to hand assisted laparoscopic surgery because the operative field could not be secured due to the massive amount of perirenal fat [14].

Based on previous reports and our long-term experience with rLNU, we hypothesized that the amount of perirenal fat affects PRT during rLNU for UTUC. Therefore, this study aimed to investigate whether the amount of perirenal fat is a risk factor for prolonged PRT during rLNU.

## Methods

### Patient selection

We retrospectively identified 115 patients who underwent rLNU for non-metastatic UTUC at our institution between 2013 and 2021.

### Surgical procedure

During retroperitoneoscopic procedure of rLNU, dissection of the kidney and upper ureter was performed using four ports (one laparoscopic trocar and three instrument trocars) in the lateral position. Sequentially, a small iliac incision (Gibson incision) in the lateral position or lower abdominal midline incision in the supine position was created to retrieve the kidney and ureter and perform bladder cuff resection with a sufficient surgical margin using the extravesical approach. At our institution, we perform lymphadenectomy only in cases suspected of visible lymph node metastasis on computed tomography (CT) and lymphadenectomy is performed using laparotomy. Therefore, lymphadenectomy was not performed in this study. Additionally, our hospital is an educational institution; therefore, it employs many non-expert surgeons. However, only non-expert surgeons with adequate skills in laparoscopic surgery are allowed to serve as primary surgeons. The criteria for a non-expert surgeon to qualify as the primary surgeon include: having acted as a scopist in at least 50 laparoscopic surgery cases, possessing adequate experience in performing laparoscopic surgeries at affiliated hospitals, having over 4 years of experience in urological surgery, being certified as a urologist by the Japanese Urological Association, and receiving an endorsement from three supervisors confirming the surgeon’s capability to perform the procedure. During surgery, the supervisor is the first assistant surgeon, providing guidance and assisting in difficult or urgent situations such as rapid hemostatic maneuvers. We defined the PRT during rLNU as the time from pressured CO_2_ gas infusion to the completion of the retroperitoneoscopic procedure.

### Evaluation of variables

We collected clinical and surgical information from the medical records of the patients. This information included age, sex, body mass index (BMI), laterality and location of the main tumor, clinical T stage, hydronephrosis grade, visceral fat area (VFA), subcutaneous fat area (SFA), posterior PFT, lateral PFT, anterior PFT, presence of preoperative diagnostic ureteroscopic biopsy, quantity of preoperatively assessed ipsilateral renal arteries and veins in the hilum, and PRT during rLNU. At our institution, preoperative diagnostic ureteroscopic biopsy is not routinely performed because it has been reported that diagnostic ureteroscopic biopsy might increase the risk of IVR [15]. In addition, adjuvant intravesical chemotherapy is not administered in our institution.

### Imaging evaluation

**Fig. 1:**
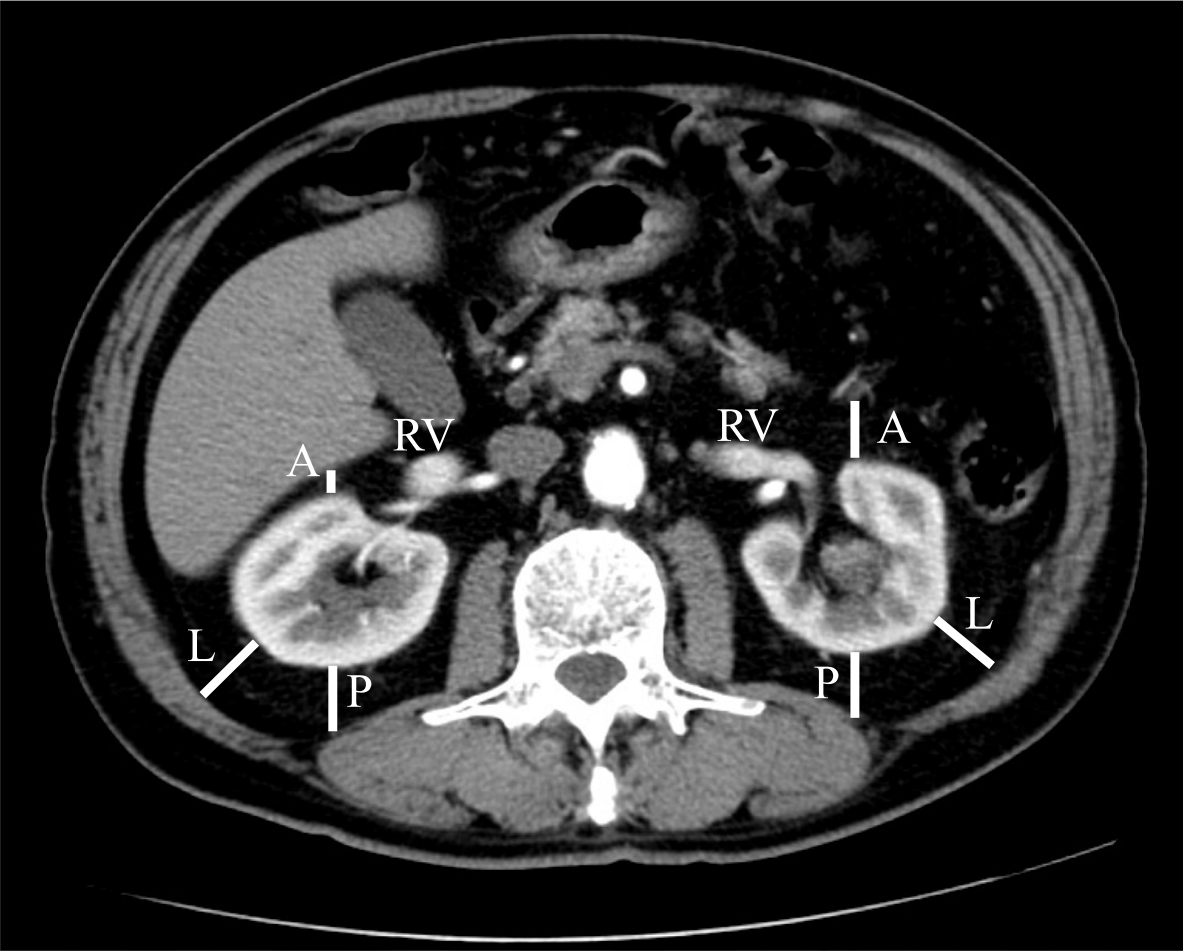
Measurements of the perirenal fat thickness (PFT) at the level of the renal vein. P, posterior PFT; L, lateral PFT; A, anterior PFT; RV: renal vein

**Fig. 2:**
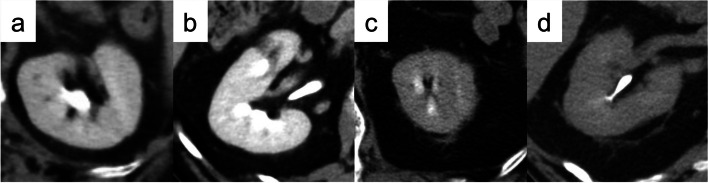
Measurements of the perirenal fat stranding. **a** No stranding. **b** Mild stranding. **c** Moderate stranding. **d** Severe stranding

**Fig. 3:**
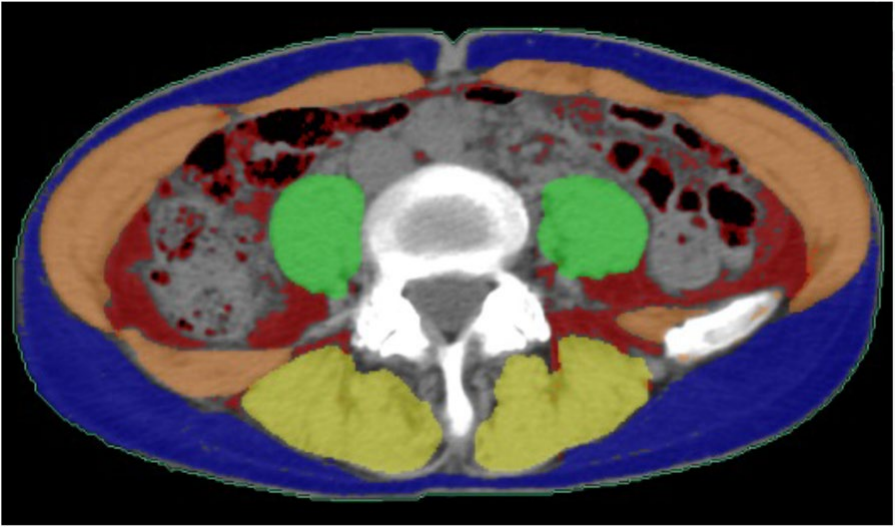
Measurements of the visceral fat area (VFA) and subcutaneous fat area (SFA). The red and blue areas represent VFA and SFA, respectively

CT findings within 3 months before surgery were evaluated by a staff radiologist (T.K.) with 21 years of experience performing urological imaging using CT. Hydronephrosis was classified as grades 0 to 4 according to the classification of Cho et al. [16]. Cases without calix or pelvic dilation were classified as grade 0, cases with pelvic dilation only as grade 1, cases accompanying mild calix dilation as grade 2, cases with severe calix dilation as grade 3, and those with calix dilation accompanied by renal parenchyma atrophy as grade 4 [16]. The PFT was measured according to the methods of Anderson et al. and Davidiuk et al. (Fig. 1) [13, 17]. Posterior, lateral, and anterior PFTs at the level of the renal veins on preoperative CT images were measured (Fig. 1). Perirenal fat stranding was classified following the method outlined by Kim et al. (Fig. 2) [18]. Cases without fat stranding were categorized as “none”; those with a few thin visible strands were categorized as “mild”; cases with numerous thick visible bands were categorized as “severe”; and cases falling between mild and severe were categorized as “moderate.” The Mayo adhesive probability (MAP) score was computed based on the sum of the posterior PFT score (1 cm = 0 points, 1.1–1.9 cm = 1 point, > 2.0 cm = 2 points) and the type of perirenal fat stranding (no stranding = 0 points, mild/moderate = 2 points, severe = 3 points), following the methodology described by Davidiuk et al. [17]. The VFA and SFA at the level of the umbilicus on preoperative images were measured using a dedicated workstation with Synapse Vincent software (Fujifilm Co. Ltd., Tokyo, Japan) (Fig. 3). Additionally, the ipsilateral renal arteries and veins on CT images were measured preoperatively.

### Endpoint of the present study

The primary endpoint of this study was the preoperative risk factors for prolonged PRT during rLNU.

### Statistical analysis

Statistical analyses were performed using IBM SPSS Statistics version 29 (IBM, Armonk, NY, USA). Statistical significance was set at *p* < 0.05. Univariate and multivariate analyses were performed using a logistic regression model to determine independent preoperative factors that predict prolonged operative times during rLNU. The cutoff value for PRT during rLNU was established based on the median of 115 patients. Additionally, cutoff values for BMI, VFA, SFA, posterior PFT, lateral PFT, and anterior PFT were determined using sensitivity and specificity levels derived from the area under the curve (AUC) of the receiver operating characteristic (ROC) curve. These values were calculated using the Youden Index formula based on the presence or absence of prolonged PRT. Age (75 years or older vs. younger than 75 years), sex, BMI, laterality, main tumor location (ureter vs. renal pelvis), tumor size (≥ 3 cm vs. < 3 cm), clinical T stage (≥ 3 vs. < 3), hydronephrosis (grade ≥ 3 vs. grade < 3), VFA, SFA, posterior PFT, lateral PFT, anterior PFT, presence or absence of perirenal fat stranding, MAP score (≥ 3 vs. ≤ 2), presence or absence of diagnostic ureteroscopic biopsy, and the quantity of ipsilateral renal arteries and veins (2 vs. ≥ 3) were assessed to identify independent preoperative factors that predict prolonged PRT during rLNU.

## Results

### Patient population

Table 1 shows the characteristics of patients who underwent rLNU. The median (interquartile [IQR], 25th–75th) values of the BMI, VFA, SFA, posterior PFT, lateral PFT, and anterior PFT were 22.7 kg/m^2^ (IQR, 20.4–25.0), 111 cm^2^ (61–150), 126 cm^2^ (102–165), 10 mm (5–15), 16 mm (8–22), and 5 mm (3–10), respectively. During this study, 13 surgeons including 3 expert surgeons performed rLNU. The median PRT and total operative time of rLNU were 182 mm (IQR, 155–230) and 341 min (IQR, 295–374), respectively.

**Table 1:** Characteristics of 115 patients who underwent rLNU

Variables		
Age, years	Median (IQR)	75 (68–79)
Sex	Male	82 (71.3)
	Female	33 (28.7)
BMI, kg/m^2^	Median (IQR)	22.7 (20.4–25.0)
Laterality	Right	59 (51.3)
	Left	56 (48.7)
Main tumor location	Ureteral	60 (52.2)
	Renal pelvic	55 (47.8)
Tumor size, cm	≤ 3	77 (67.0)
	> 3	38 (33.4)
Clinical T stage	≤ 2	75 (65.2)
	≥ 3	40 (34.8)
Hydronephrosis grade	0	59 (51.3)
	1	14 (12.2)
	2	21 (18.3)
	3	12 (10.4)
	4	9 (7.8)
VFA, cm^2^	Median (IQR)	111 (61–150)
SFA, cm^2^	Median (IQR)	126 (102–165)
Posterior PFT, mm	Median (IQR)	10 (5–15)
Lateral PFT, mm	Median (IQR)	16 (8–22)
Anterior PFT, mm	Median (IQR)	5 (3–10)
Perirenal fat stranding type	None/ mild or moderate/ severe	66 (57.4)/ 44 (38.3)/5 (4.3)
MAP score	≤ 2	78 (77.2)
	≥ 3	37 (32.8)
Diagnostic ureteroscopic biopsy	Yes / No	33 (28.7)/ 82 (71.3)
Renal arteries and veins, no	Median (IQR)	2 (2–3)
Pneumoperitoneum time, min	Median (IQR)	182 (155–230)
Total operative time, min	Median (IQR)	341 (295–374)

*BMI* body mass index, *IQR* interquartile range, *rLNU* retroperitoneal laparoscopic nephroureterectomy, *VFA* visceral fat area, *SFA* subcutaneous fat area, *PFT* perirenal fat thickness, *MAP* Mayo adhesive probability

Data are presented as *n* (%) or median (IQR) unless otherwise indicated

### Evaluation of preoperative factors associated with PRT during rLNU

**Fig. 4:**
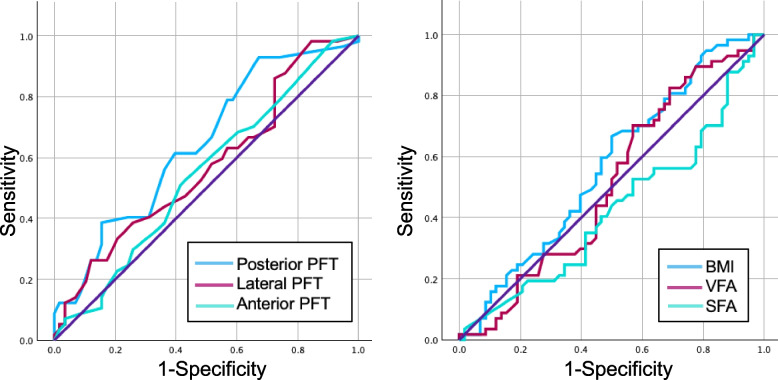
Receiver operating characteristic (ROC) curve of variables. PFT, perirenal fat thickness

We defined the cutoff value of PRT during rLNU as 182 min. Figure 4 demonstrates the ROC curves of the BMI, VFA, SFA, posterior PFT, lateral PFT, and anterior PFT. Table 2 presents the results of the ROC curve analysis. The AUC for posterior PFT was 0.646 (*p* = 0.004, 95% confidence interval [CI] 0.545–0.746). The cutoff values for BMI, VFA, posterior PFT, lateral PFT, and anterior PFT were defined as 21.9 kg/m^2^, 92 cm^2^, 15 mm, 24 mm, and 6 mm, respectively. The median was used as the cutoff value for SFA because the AUC for SFA was < 0.5. Univariate logistic regression analysis revealed that a posterior PFT ≥ 15 mm (*p* = 0.0067) and MAP score ≥ 3 (*p* = 0.0262) were risk factors for prolonged PRT during rLNU. Multivariate logistic regression analysis revealed that a posterior PFT ≥ 15 mm (*p* = 0.0410; odds ratio [OR], 2.72; 95% CI, 1.04–7.08) was an independent risk factor for prolonged PRT during rLNU (Table 3).

**Table 2:** ROC curves analysis of variables

	AUC	Cut off	Sensitivity	Specificity	Youden Index	95% CI	*P*-value
BMI	0.567	21.9	0.667	0.5	0.167	0.462–0.672	0.214
VFA	0.511	92	0.702	0.431	0.133	0.403–0.618	0.847
SFA	0.425	41	0.035	0.983	0.034	0.320–0.530	0.161
Posterior PFT	0.646	15	0.386	0.845	0.231	0.545–0.746	*0.004
Lateral PFT	0.544	24	0.263	0.879	0.142	0.469–0.678	0.169
Anterior PFT	0.574	6	0.526	0.569	0.095	0.439–0.650	0.411

*AUC* area under the curve, *CI* confidence interval, *BMI* body mass index, *VFA* visceral fat area, *SFA* subcutaneous fat area, *PFT* perirenal fat thickness

^*^p < 0.05

**Table 3:** Univariate and multivariate logistic regression analyses of preoperative risk factors for prolonged PRT during rLNU using the median as cutoff value

	PRT			
	Univariate	Multivariate		
	*P*	OR	95% CI	*P*
Age younger than 75 years	0.2257			
Male sex	0.3333			
BMI ≥ 21.9 kg/m^2^	0.6314			
Right tumor	0.7777			
Ureteral tumor	0.3061			
Tumor size ≥ 3 cm	0.3436			
Clinical T stage ≥ 3	0.6457			
Hydronephrosis grade ≥ 3	0.8436			
VFA ≥ 92 cm^2^	0.1409			
SFA ≥ 127 cm^2^	0.4008			
Posterior PFT ≥ 15 mm	*0.0067	2.72	1.04–7.08	*0.0410
Lateral PFT ≥ 24 mm	0.0572			
Anterior PFT ≥ 6 mm	0.3072			
Presence of perirenal fat stranding	0.1636			
MAP score ≥ 3	*0.0262	1.76	0.72–4.27	0.2147
Presence of diagnostic biopsy	0.2773			
Renal vessels, *n* ≥ 3	0.4918			

*PRT* pneumoretroperitoneum time, *rLNU* retroperitoneal laparoscopic nephroureterectomy, *OR* odds ratio, *CI* confidence interval, *BMI* body mass index, *VFA* visceral fat area, *SFA* subcutaneous fat area, *PFT* perirenal fat thickness, *MAP* Mayo adhesive probability

^*^p < 0.05

## Discussion

In the present study, multivariate analyses using a logistic regression model revealed that thick posterior PFT was the only independent risk factor for prolonged PRT during rLNU (Tables 2 and 3). In contrast, indicators of obesity, such as BMI, VFA, and SFA, were not significant risk factors for prolonged PRT during rLNU. This is the first study to reveal that the PFT is associated with the PRT during rLNU. Anderson et al. reported that the amount of perirenal fat, especially thats of anterior perirenal fat, rather than the amount of intraperitoneal fat, was correlated with the operative time during laparoscopic donor nephrectomy using the peritoneal approach [13]. Furthermore, they speculated that perirenal fat obscured anatomic landmarks, thus making it difficult to identify the location of the renal vessels and their branches, whereas intraperitoneal fat had little influence on surgery [13]. The retroperitoneal approach to laparoscopic renal surgery has the advantage of avoiding thick subcutaneous fat and visceral fat in patients with obesity [19, 20]. However, the narrow working space associated with the retroperitoneal approach during laparoscopic renal surgery requires a skilled and experienced surgeon. Additionally, thick perirenal fat poses the difficulty of dissecting the renal hilum, including the renal vessels, which is a process that requires careful manipulation during rLNU. Therefore, patients with thick posterior perirenal fat require careful renal hilum manipulation in a narrow surgical field and appropriate traction of the posterior perirenal fat covering the renal hilum to secure the surgical field. Based on published research and our own experience, we speculate that a greater posterior PFT prolongs the PRT during rLNU.

Posterior PFT measurements are easily performed using plain CT images because a dedicated workstation is not required; therefore, they can be routinely performed in clinical practice. Using these measurements, prolonged PRT during rLNU can be predicted. Based on the results of this study, rLNU should be performed by an experienced surgeon and not by a trainee or non-expert surgeon, when patients have thick posterior PFT on preoperative CT images and are at higher risk for prolonged PRT. Moreover, when patients have very thick posterior PFT on preoperative CT images and are at higher risk for prolonged PRT, selecting transperitoneal approach to surgery or open surgery should be considered. It has been reported that a single early intravesical chemotherapy cycle using mitomycin C or pirarubicin after NU decreases the risk of IVR [21, 22]. Adjuvant intravesical chemotherapy might be considered for cases with prolonged PRT during rLNU.

While the influence of a thick PFT on surgical and oncological outcomes remains uncertain, several studies have reported negative effects of thick PFT on these outcomes in certain types of cancer [10–12, 23]. Generally, oncological surgeries in patients with a thick PFT may lead to increased postoperative complications and poorer oncological results. Therefore, it is advisable to complete procedures swiftly and safely in such cases. Expert surgeons should ideally handle the entire procedure from start to finish for patients with thick PFT, especially in those with low surgical tolerance, such as older adults or those with multiple complications. Conversely, cases with thin PFT and high tolerance may be suitable for non-expert surgeons.

This study had several limitations. UTUC is relatively uncommon, and this study was conducted at a single institution; therefore, the cohort was small. The characteristics of the facility, including the availability of surgical equipment such as energy devices and retractors, as well as facility-specific surgical techniques, could impact the insufflation time. These facility-related factors might affect the applicability of the present study findings. To address these limitations, future prospective studies involving multiple institutions and larger cohorts are needed. Because this study was a retrospective analysis, and because our institution is an educational institution, selection bias for surgeons may have occurred. In this study, 10 non-expert surgeons with adequate technical skills but not yet experts performed rLNU. However, three expert surgeons supervised all rLNU procedures. Therefore, we believe that the selection of surgeons did not affect the ranking of the operating times significantly. However, previous reports indicate that factors prolonging operative time in laparoscopic surgery may vary between expert and non-expert surgeons [24]. Thus, future studies in non-educational settings are warranted. Additionally, this study exclusively involved Japanese patients whose body size and other patient characteristics may differ from those in other countries. Therefore, further investigations in countries beyond Japan are necessary. Currently, lymphadenectomy is recommended for pathological T ≥ 2 UTUC. However, lymphadenectomy was not performed in this study because there are several discrepancies between the clinical T stage and pathological T stage, and there are technical issues with rLNU. However, lymphadenectomy only in cases suspected of visible lymph node metastasis on imaging might result in worse oncological outcome. We need to perform lymphadenectomy for clinical T ≥ 2 UTUC and improve the accuracy of diagnostic imaging for clinical T ≥ 2 UTUC staging and lymph node dissection, as well as technical skills to perform laparoscopic lymphadenectomy. In the future, analysis including cases with laparoscopic lymphadenectomy is required.

## Conclusions

Thick posterior PFT is a preoperative risk factor for prolonged PRT during rLNU. For patients with UTUC and thick posterior PFT, surgeons should develop optimal surgical strategies, including the selecting an expert surgeon as a primary surgeon and the selecting transperitoneal approach to surgery or open surgery.

## Data Availability

The datasets used and/or analyzed in the current study are available from the corresponding author upon reasonable request.

## Abbreviations

AUC: Area under the curve
BMI: Body mass index
CI: Confidence interval
CT: Computed tomography
IQR: Interquartile range
IVR: Intravesical recurrence
LNU: Laparoscopic nephroureterectomy
MAP: Mayo adhesive probability
NU: Nephroureterectomy
OR: Odds ratio
PFT: Perirenal fat thickness
PRT: Pneumoretroperitoneum time
rLNU: Retroperitoneal laparoscopic nephroureterectomy
ROC: Receiver operating characteristic
SFA: Subcutaneous fat area
UTUC: Upper tract urothelial cancer
VFA: Visceral fat area
